# Role of interleukin-18 in mediating the impacts of celiac disease on osteoporosis: a Mendelian randomization study

**DOI:** 10.3389/fimmu.2024.1453657

**Published:** 2024-10-09

**Authors:** Jie Xiang, Xiaoyu Zheng, Lan Luo, Xiaoqiang Yang

**Affiliations:** ^1^ Department of Gastroenterology, The Central Hospital of Enshi Tujia and Miao Autonomous Prefecture, Enshi, China; ^2^ Department of Anesthesiology, The Central Hospital of Enshi Tujia and Miao Autonomous Prefecture, Enshi, China

**Keywords:** celiac disease, osteoporosis, inflammation, Mendelian randomization, mediation

## Abstract

**Background:**

Extensive observational data suggest a link between celiac disease (CeD) and osteoporosis, but the causality and mediating mechanism remain undetermined. Herein, we performed a Mendelian randomization (MR) study to address these concerns.

**Methods:**

We obtained the summary-level statistics for CeD from a large genome-wide association study (GWAS) comprising 4,533 cases and 10,750 controls of European ancestry. The GWAS data for osteoporosis-related traits and inflammatory cytokines were derived from the UK Biobank, FinnGen, IEU OpenGWAS database, or GWAS catalog. Two-sample MR with the inverse variance-weighted methods were employed to evaluate the genetic association between CeD and osteoporosis-related traits. The potential inflammatory mediators from CeD to osteoporosis were explored using two-step mediation analyses.

**Results:**

The primary MR analyses demonstrated causal associations between genetically predicted CeD and osteoporosis (odds ratio [OR]: 1.110, 95% confidence interval [CI]: 1.043–1.182, *p*=0.001), total body bone mineral density (β: -0.025, *p*=0.039), and osteoporotic fracture (OR: 1.124, 95% CI: 1.009–1.253, *p*=0.034). Extensive sensitivity analyses consolidated these findings. Among the candidate inflammatory cytokines, only interleukin-18 was observed to mediate the effects of CeD on osteoporosis, with an indirect OR of 1.020 (95% CI: 1.000–1.040, *p*=0.048) and a mediation proportion of 18.9%. The mediation effects of interleukin-18 could be validated in other datasets (OR: 1.015, 95% CI: 1.001–1.029, *p*=0.041). Bayesian colocalization analysis supported the role of interleukin-18 in osteoporosis.

**Conclusion:**

The present MR study reveals that CeD is associated with an increased risk of developing osteoporosis, which may be partly mediated by upregulation of interleukin-18.

## Introduction

Celiac disease (CeD) is a chronic immune-mediated enteropathy triggered by intolerance to gluten proteins in genetically predisposed subjects (1). Approximately 0.7–1.4% of the general population worldwide are affected by this illness, and the prevalence appears to be raising over time (2, 3). Within the small bowel of individuals with CeD, gluten digestion products (e.g., omega-5-gliadin) penetrating into the lamina propria can directly, or after deamination by tissue transglutaminases in the submucosa, activate the innate immune system (4). This mechanism induces an abnormal inflammatory cascade followed by damage to the structure and function of intestinal tissue (5). Although CeD primarily attacks the small intestine, it is increasingly recognized as a systemic autoimmune disorder that may present with a diverse of extraintestinal comorbidities, such as type 1 diabetes, autoimmune liver disease, and psoriasis (6).

Osteoporosis is a skeletal disorder featured by low bone mineral density (BMD) and deterioration of bone microarchitecture, with a consequent increase in susceptibility to bone fragility or fractures (7). As a global prevalent disease in the elderly, osteoporosis causes more than 8.9 million pathological fractures each year, casting a heavy economical burden to many regions (8). Nearly 14.4% of patients with CeD are suffering from osteoporosis (9). Accumulating data from population-based studies have also demonstrated that CeD was associated with reduced BMD and might represent an independent risk factor for osteoporotic fracture (10). However, whether these relationships are causal or driven by shared environmental factors remains undetermined, mainly owning to the inherent drawbacks of observational study designs. Traditional observational studies are susceptible to reverse causality as they are always problematic to determine which of two associated variables is the cause; and, confounding bias is more difficult to control for because it is mainly due to social, behavioral, or physiological factors that are difficult to measure and deal with (11). Therefore, previous observational data are insufficient to establish causal insights between CeD and osteoporosis.

Additionally, the etiopathology of osteoporosis in CeD remains largely under-investigated. The osteoporosis may be caused by malabsorption of calcium or vitamin D, but also other factors such as chronic inflammation in CeD can exert an crucial role (12). CeD is characterized by an intestinal Th1 response to dietary gluten, presenting with hypersecretion of proinflammatory proteins in the damaged mucosa or serum, particularly interferon (IFN)-ϒ, tumor necrosis factor (TNF), interleukin (IL)-1, and IL-6 (13). These cytokines are implicated in bone metabolism as they regulate the differentiation and activation of osteoblasts or osteoclasts (14). IL-18, also known as an IFN-ϒ inducing factor, are produced linked to gluten intake and associated with Th1 activity in CeD (15). Recent reports have shown that IL-18 maintain a long-standing inflammation status in CeD patients (16) and can up-regulate the expression of key osteoclastogenic regulators (17), pointing towards a mediation role in the CeD-induced osteoporosis.

Mendelian randomization (MR) is an epidemiological strategy widely used to strengthen the causal inference by employing single nucleotide polymorphisms (SNPs) as unbiased instrumental variables (IVs) for exposures (18). Because genetic variants are allocated randomly during gametogenesis and would not be modified by acquired factors, MR procedure furnishes several advantages over observational designs: 1) it ensures the temporality of exposure and outcome, preventing reverse causation; 2) it minimizes the impact of residual or unmeasured confounding factors; 3) it reflects the long-term risk estimates of exposure, as the IVs remain valid throughout a lifetime (19). MR can thus represent an analogue to randomized controlled trials that utilizes genetic variation as the method for randomization ultimately providing causal inferences. In this study, we applied the MR approach to explore the causal association between CeD and osteoporosis-related traits, and simultaneously examined the mediating relationship of inflammatory cytokines between CeD and osteoporosis.

## Methods

### Study design

**Figure 1:**
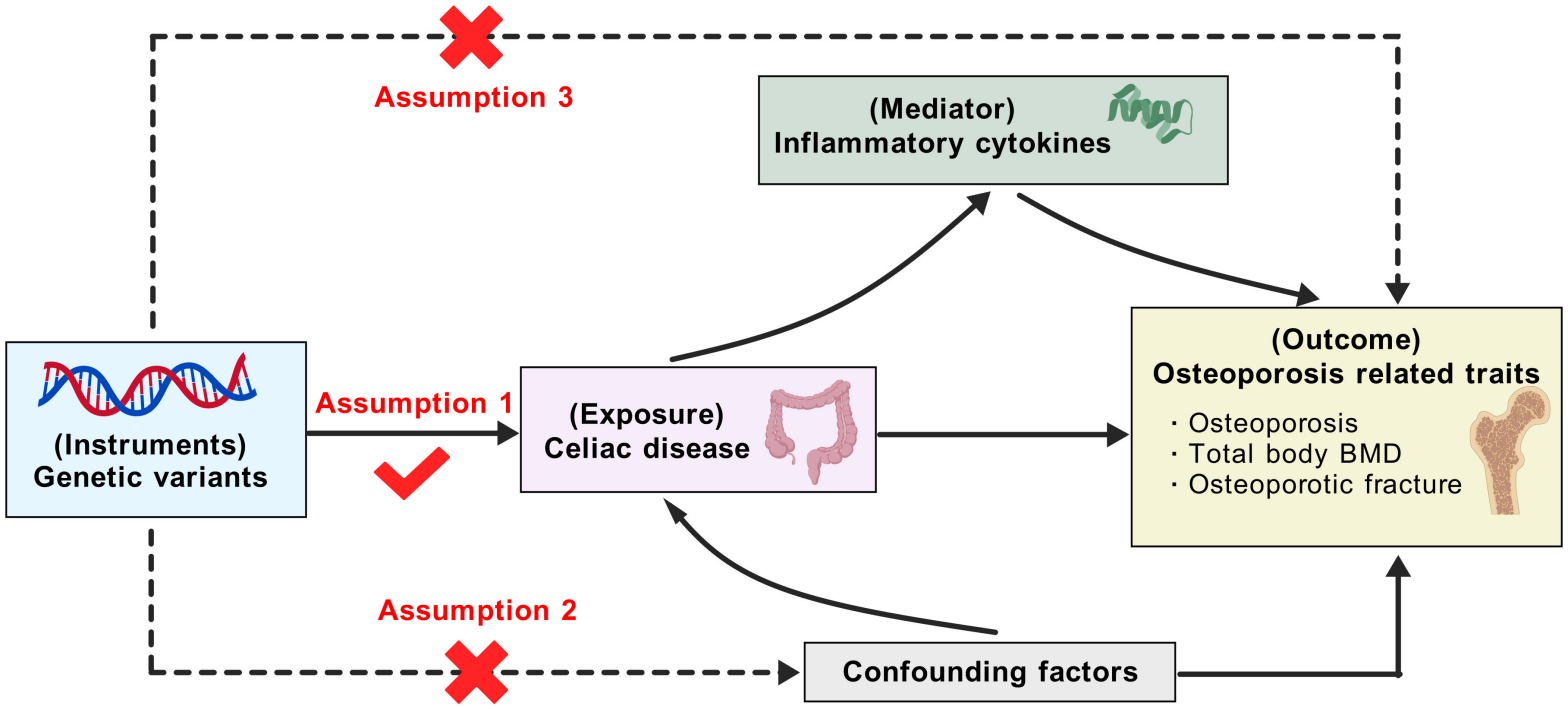
Outline of this Mendelian randomization study. BMD, bone mineral density (the figure was created with BioGDP.com). Based on the Mendelian randomization assumptions, the genetic variants are assumed to affect osteoporosis through celiac disease only, not through confounding factors or directly associated with osteoporosis. Then, the mediation effects of potential inflammatory cytokines between celiac disease and osteoporosis were examined.

The overall design of this work was shown in **Figure 1**. Briefly, we first employed two-sample MR methods to assess the associations of CeD with osteoporosis-related traits, including osteoporosis, total body BMD, and osteoporotic fracture. Then, two-step MR strategies were leveraged to explore the mediating effects of inflammatory candidates in the associations. MR analyses can provide unbiased causal inference if the selected IVs satisfy the following three assumptions: 1) the IVs must be strongly associated with exposures; 2) the IVs should be free of confounding factors; 3) the IVs affect the outcomes solely through the exposures. This study complies with the Strengthening the Reporting of Observational Studies in Epidemiology Using Mendelian Randomization (STROBE-MR) statement (seeing the checklist in **Supplementary Table S1**).

### Data source

Our MR study utilized publicly accessible data from genome-wide association studies (GWAS) or databases (**Supplementary Table S2**). The original articles have provided the ethical clearance and consent to participates, thus there was no need for additional approvals. The summary-level genetic statistics for CeD were derived from a GWAS study involving 4,533 cases and 10,750 controls of European ancestry (IEU OpenGWAS ID: ieu-a-276) (20). The summary-level GWAS data pertaining to osteoporosis and osteoporotic fracture were acquired from the UK Biobank (21) and FinnGen (release 10) (22) consortium, respectively. The former consisted of 6,484 osteoporosis individuals and 401,279 controls, and the latter comprised 1,822 cases and 311,210 controls. The genetic statistics for total body BMD were sourced from a GWAS meta-analysis of 30 cohorts totaling 56,284 European participates (23).

Based on recent literature reviews (13, 24), we examined the mediating effects of nine inflammatory proteins in the CeD-osteoporosis association, including IL-1α, IL-1β, IL-6, IL-10, IL-12, IL-18, TNF-α, TNF-β, and IFN-γ. The GWAS summary statistics correlated with these cytokines were retrieved from three meta-analyses (25–27) of pQTL, with the number of participates ranging from 3,309 to 21758 as described in **Supplementary Table S2**. If a cytokine was measured in at least two studies, we used the pQTL data with the largest sample size.

### Instrumental variable selection

For CeD, we extracted SNPs that were highly associated with this illness at a genome-wide significance level (*p*<5×10^-8^). For each inflammatory protein, cis-pQTLs strongly correlated with the protein level (*p*<5×10^-8^) were collected. Cis-pQTLs were defined as the pQTLs locating at ±1 Mb from the encoding gene. To identify independent IVs, the selected genetic variants were pruned through linkage disequilibrium (LD) R^2^<0.001 within 10-Mb windows, using the 1000 Genomes Project of European ancestry as reference panel (28). Confounding biases were minimized by removing any pleiotropic SNPs using the LDTrait tool (29), and SNPs that directly influenced the outcomes (*p*<5×10^-8^) were excluded. During MR harmonization process, SNPs not present in the outcome GWAS data or those being palindromic were further discarded. The remaining genetic variants were screened out as IVs, and their strengths were assessed using the *F* statistics, calculated as dividing the square of β coefficient by the square of standard error.

### Statistical analysis

In the primary MR analyses, we used the inverse-variance weighted (IVW) method or the Wald ratio to estimate the causal effects of exposures on outcomes. Heterogeneity was detected using Cochrane’s Q-test, with *p*>0.05 indicating no substantial heterogeneity. We applied the MR-Egger intercept test to evaluate potential horizontal pleiotropy, where the difference between the intercept of the MR-Egger regression and zero were tested (30). The IVW approach has the highest statistical power but may be biased when pleiotropy exists; therefore, we further introduced the MR-Egger with bootstrapping (30) and the weighted median (31) methods for reanalysis. The MR-Egger approach can provide genetic estimates corrected for pleiotropy because it allows nearly all of SNPs to be horizontally pleiotropic; and, the weighted median can generate unbiased causal estimates when more than half of the IVs are valid. MR-pleiotropy residual sum and outlier (MR-PRESSO) test was also employed to detect the outliers involved in horizontal pleiotropy and produce the corrected results (32). Leave-one-out analyses were performed to assess the influence of individual SNPs on the MR results.

Two-step MR was conducted to identify the potential inflammatory proteins that mediate the effects of CeD on osteoporosis. In the first step, we performed MR analyses with the IVW method to examine the causal effects of CeD on the nine inflammatory proteins. The proteins that passed the significance threshold (*p*<0.05) were entered into the second step, in which their causal effects on the risk of osteoporosis were investigated. The MR estimates of CeD on osteoporosis, CeD on each protein, and each protein on osteoporosis were recorded as β0, β1, and β2, respectively. For an inflammatory cytokine with both β1 and β2 being significant, we conducted mediation analysis to further elucidate whether it could mediate the effect of CeD on osteoporosis risk. Indirect effect, which refers to the effect of CeD on osteoporosis through the inflammatory cytokine, was computed using the “Product of coefficients” method (33) (β1×β2). We also estimated the corresponding proportion of mediation as the indirect effect divided by the total effect (β1×β2/β0). The 95% confidence intervals (CIs) were obtained from the delta method. The MR-Steiger test was performed to validate the directionality of causal relationships between CeD, inflammatory mediator, and osteoporosis.

For a cytokine with significant mediating effect, Bayesian colocalization analysis (34) was conducted to reinforce the MR assumption in osteoporosis. This approach calculates the posterior probabilities (PP) for five hypothesis testing: H0 (no causal variants), H1 (causal variant for the cytokine only), H2 (causal variant for osteoporosis only), H3 (separate causal variants for the cytokine and osteoporosis), and H4 (shared causal variant for the cytokine and osteoporosis). We selected all SNPs located within ±100 kb around the lead cis-pQTL of the cytokine encoding gene for colocalization. PP.H4 > 0.5 was considered evidence of colocalization, implying that colocalization is more likely than any other situations combined.

All statistical analyses were implemented using R version 4.1.0 (The R Foundation for Statistical Computing, Vienna, Austria) software. The “TwoSampleMR” (version 0.5.6), “RMediation” (version 1.2.2), and “coloc” (version 5.2.3) R packages were used for the main analyses. A two-sided *p* value of < 0.05 was deemed as of significance.

## Results

### Instrument variables for CeD

The correlation analyses and LD clumping generated 12 independent genetic variants that were strongly associated with CeD. **Supplementary Table S3** summarized the details of these IVs for CeD. Of them, rs653178 was interfered with other traits such as smoking and diabetes; therefore, we removed this pleiotropic SNP from the subsequent MR analyses of the CeD-osteoporosis association. The *F* statistics ranged from 29 to 101 for the selected instrumental SNPs, signifying that our MR analyses were robust against weak instrument bias.

### CeD and osteoporosis-related traits

**Figure 2:**
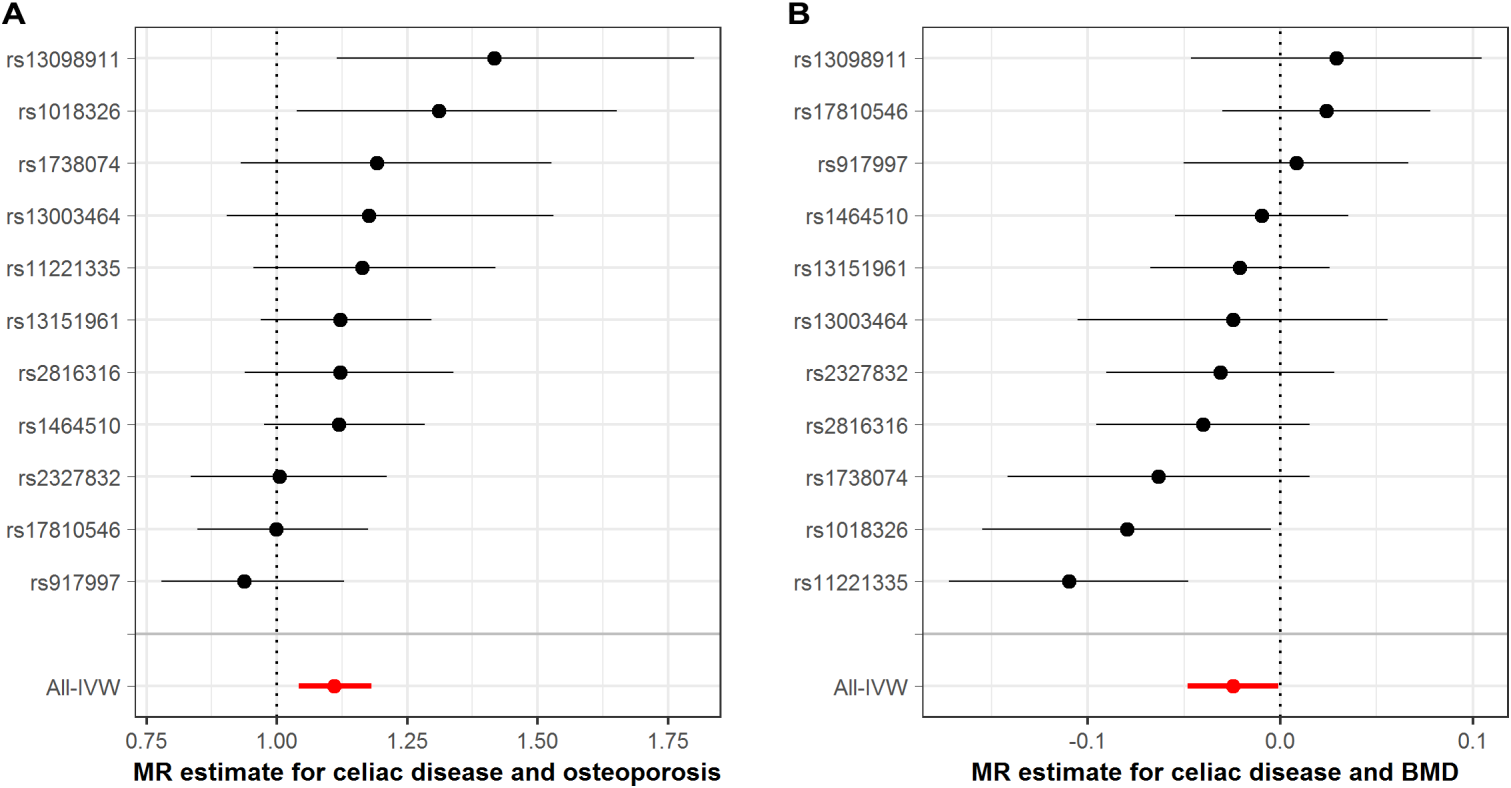
Association between genetically proxied celiac disease with osteoporosis-related traits. **(A)**, estimates for celiac disease and osteoporosis risk; **(B)**, estimates for celiac disease and BMD. BMD, bone mineral density; IVW, inverse-variance weighted; MR, Mendelian randomization. The dots represent the odds ratios of estimates, and the bars represent the corresponding 95% confidence intervals.

The MR associations between CeD and osteoporosis-related phenotypes were exhibited in **Figure 2** and **Supplementary Table S4**. The IVW-MR analyses indicated that genetically instrumented CeD was related to higher risks of developing osteoporosis (odds ratio [OR]: 1.110, 95% CI: 1.043–1.182, *p*=0.001; **Figure 2A**) and osteoporotic fracture (OR: 1.124, 95% CI: 1.009–1.253, *p*=0.034). Meanwhile, we found that genetic liability to CeD could decrease total body BMD (β: -0.025, *p*=0.039; **Figure 2B**). The results from the MR-Egger and the weighted median methods, which provide adjustments for potential pleiotropic effects, were directionally consistent with the IVW estimates albeit with noticeably wider CIs (**Supplementary Table S4**). The Cochran Q-test documented no significant heterogeneity for the above findings (**Supplementary Table S5**). There were no evidence of horizontal pleiotropy from the MR-Egger regression intercept test and the MR-PRESSO Global test (**Supplementary Table S5**), implying that the IVW estimates were credible. Leave-one-out sensitivity analyses indicated that our MR results were not driven by a single instrumental SNP (**Supplementary Table S6**), further reinforcing the robustness of the MR conclusions.

### Mediation effects of inflammatory cytokines

**Figure 3:**
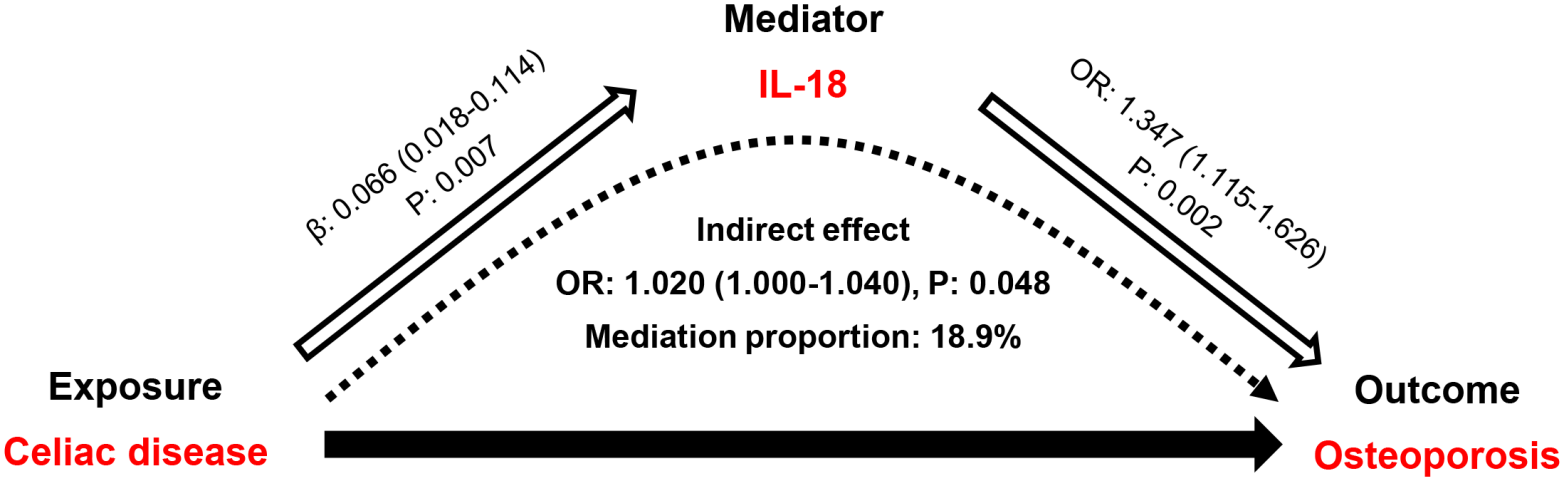
Mediation effect of IL-18 in the association between celiac disease and osteoporosis. IL-18, interleukin-18; OR, odds ratio. The indirect effect refers to the effect of celiac disease on osteoporosis through IL-18.

Among the nine candidate inflammatory cytokines, we observed that only IL-6 and IL-18 could be affected by genetically surrogated CeD. The results showed that CeD was linked to an increased expression of plasma IL-6 (β: 0.038 95% CI: 0.002–0.073, *p*=0.037) and IL-18 (β: 0.066, 95% CI: 0.018–0.114, *p*=0.007; **Supplementary Table S7**). However, there was no significant cis-pQTLs identified for IL-6 in the pQTL datasets; therefore, we only retained IL-18 for the following analyses. By using one cis-pQTL (rs5744249, located in the intron of IL18 gene) as the instrument, we found that plasma level of IL-18 was positively associated with osteoporosis risk (OR: 1.347, 95% CI: 1.115–1.626, *p*=0.002). These findings indicated that IL-18 might serve as a mediator from CeD to osteoporosis. As expected, the mediation analysis suggested that the indirect effect of IL-18 was 1.020 (95% CI: 1.000–1.040, *p*=0.048), with a mediation proportion of 18.9% in the CeD-osteoporosis association (**Figure 3**). The MR-Steiger test confirmed the causal directions from CeD to IL-18 and from IL-18 to osteoporosis (**Supplementary Table S8**). To validate the mediation effects, we analyzed another two pQTL datasets (25, 26) for IL-18 (seeing **Supplementary Table S2**). We first combined the MR estimates (β1 or β2) of IL-18 from each pQTL dataset using random-effect meta-analysis and then repeated the aforementioned mediation analyses. The results also demonstrated that IL-18 could mediate the effect of CeD on osteoporosis (indirect effect, OR: 1.015, 95% CI: 1.001–1.029, *p*=0.041; **Supplementary Table S9**), with a mediation proportion of 14.0%.

### Colocalization between IL-18 and osteoporosis

**Figure 4:**
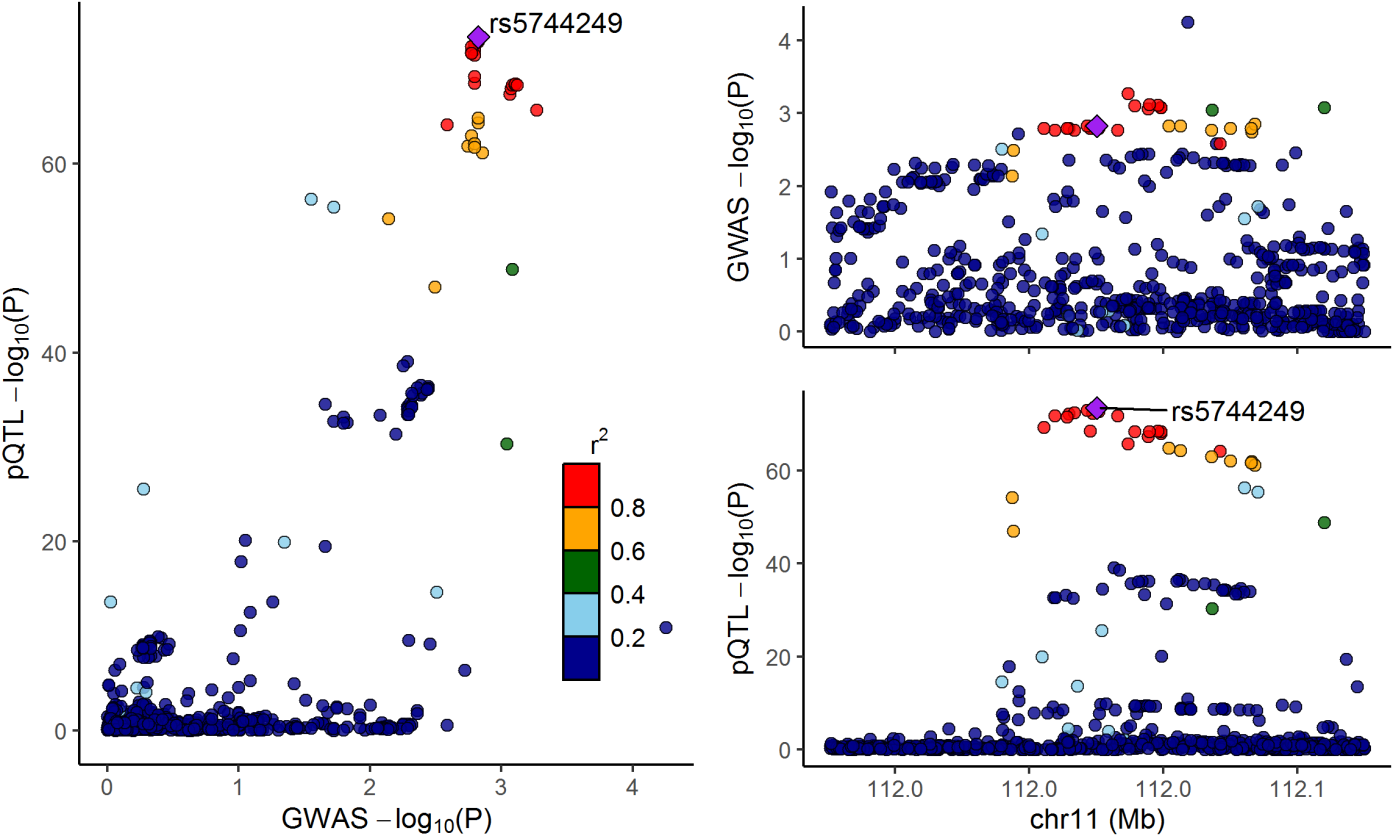
LocusCompare plot for the colocalization between interleukin-18 pQTLs and osteoporosis. The dots represent the genetic variants at chromosome 11, with color indicating the magnitude of the p-value. The purple rhombus stands for the shared causal variant, rs5744249.

Considering that the above analysis identified IL-18 as a mediator between CeD and osteoporosis, we further performed colocalization analyses to investigate whether the cis-pQTLs of IL-18 shared the same casual variants with osteoporosis. The results provided evidence for colocalization between the protein expression of IL-18 and osteoporosis (PP.H4 = 0.56, **Figure 4** and **Supplementary Table S10**). This finding reinforces the aforementioned MR results and underlines that IL18 may represent a potential therapeutic target in osteoporosis.

## Discussion

CeD is a chronic immuno-inflammatory disease with a broad spectrum of extraintestinal comorbidities. In the present MR investigation, we found that genetic predisposition to CeD could increase the risks of osteoporosis and osteoporotic fracture and reduce total body BMD. Additionally, plasma IL-18 levels appeared to play an important role in mediating the relationship between CeD and osteoporosis. The colocalization analysis supported the connection between IL-18 and osteoporosis.

CeD has been recognized for decades as a secondary cause of osteoporosis, but most of the relevant data were sourced from observational studies. For example, a nationwide cohort study of 103,361 individuals demonstrated that CeD was an independent risk factor for developing osteoporosis and osteoporotic fractures, both before or after the diagnosis (35). A meta-analysis of prospective cohort studies indicated that CeD at baseline conferred a 30% increase in the risk of any fractures and a 69% increase in the risk of hip fracture (36). As mentioned above, MR is effective in avoiding the bias from observational nature including residual confounding and reverse causation. The present MR analyses revealed causal links of CeD with osteoporosis-related traits, reinforcing the conclusion from conventional epidemiological studies. Our results further highlight the importance of BMD monitoring in the clinical management of patients with CeD, as it proposed in recent guidelines (37, 38).

The mechanisms underlying osteoporosis in CeD remain incompletely understood. One of the theories assumes an impaired intestinal absorption of vitamin D and calcium in CeD, leading to secondary hyperparathyroidism and subsequent osteoclast-mediated bone turnover (13). In symptomatic CeD, the bone loss appears directly associated with intestinal malabsorption of vitamin D, calcium, and other nutrients that are essential to bone health. However, low BMD can be observed even in patients with atypical or asymptomatic CeD at the time of diagnosis (39), raising the possibility of other determinants for the origin of osteoporosis in CeD. In recent years, accelerating evidence have emphasized the role of both local and systemic inflammation in the pathological process of CeD-related bone loss, characterized by a chronic increase in both mucosal and circulating pro-inflammatory cytokines (40). Pro-inflammatory cytokines may imbalance the receptor activator of nuclear kappa-B ligand (RANKL)/osteoprotegerin (OPN) pathway by lowering the OPN to RANKL ratio thus favoring osteoclastogenesis (41). Previous studies have also shown a decreased OPG/RANKL ratio under the condition of CeD, which was positively correlated with BMD at the spine (42). Among the inflammatory cytokines, we observed that IL-18 might mediate the effect of CeD on osteoporosis. IL-18 was reported to be activated at a post-translational level in patients with CeD (15), which in turn sustained a long-standing intestinal inflammation (16). As a powerful inflammatory cytokine, IL-18 can facilitate osteoclast differentiation by boosting inflammatory response via inducing the secretion of critical inflammatory factors (e.g., IFN-γ and TNF -α) as well as acting on T lymphocytes (43). IL-18 also suppress the secretion of osteogenic related proteins or transcription factors, such as Wnt-10b, Runx-2, and BMP-2 (44). Overall, IL-18 seems to play a pivotal role in creating and maintaining a chronic immuno-inflammatory microenvironment that provokes osteoclasis and inhibits osteogenesis in CeD (45). Accordingly, administration of IL-18 antagonists can improve osteoporosis and reduce pro-inflammatory cytokines in ovariectomized mice (46). In this study, we found that the protein expression of IL-18 was colocalized with osteoporosis, further supporting its role in the bone mass deterioration in CeD patients.

Our findings undoubtedly supported the assessment of BMD for CeD at the time of diagnosis, but whether it should be routinely implemented in all patients, particularly in those being atypical or asymptomatic, remains debated in recent guidelines (40). This has risen the need for clinical or biochemical marker to select high-risk patients of developing bone loss. In the present study, we identified IL-18 as mediator between CeD and osteoporosis, reflecting that IL-18 measurement may help the risk classification of osteoporosis in atypical or asymptomatic CeD patients. Currently, a lifelong gluten-free diet (GFD) is still considered to be the mainstay treatment for patients with CeD and bone diseases (47). Nevertheless, some studies suggested that despite strict adherence to GFD, more than half of patients displayed low BMD and continued to experience a higher rate of osteoporosis, denoting that the persistent activation of inflammation should be considered in such residual risk (48, 49). As mentioned above, IL-18 is a dominate cytokine to maintain the long-standing inflammatory nature in CeD. Targeting IL-18 was found to be effective in experimental models of autoimmune diseases including inflammatory bowel disease (50) and rheumatoid arthritis (51), as well as to be safe in clinical patients (52). Alongside with these reports, our results indicated that IL-18 may also serve as a potential target for the prevention and treatment of osteoporosis in patients with CeD.

Several limitations should be acknowledged. First of all, although we have performed extensive analyses to reinforce the MR inferences, the horizontal pleiotropy cannot be completely eliminated. Secondly, due to the lack of relevant data, it is challenging to determine whether our MR findings are affected by potential confounding factors, such as age, gender, reproductive status, smoke, steroid exposure, and endocrine disorders. Thirdly, despite being documented as a potential mediator, the exact biological pathway though which IL-18 promotes osteoporosis in CeD remains unclear. This is a research topic and should be taken into consideration in future works. Fourthly, the original GWAS datasets only include European individuals, limiting the generalization of our results to other ethnics.

In summary, our MR research indicates that patients with CeD are at higher risks of developing osteoporosis, which may be partly mediated by the increased level of IL-18. These findings support the assessment of BMD in CeD management, and put forward that IL-18 may represent a potential target in the management of CeD-induced osteoporosis, which requires further exploration in future researches.

## Data Availability

The original contributions presented in the study are included in the article/**Supplementary Material**. Further inquiries can be directed to the corresponding authors.
